# Determinants that attract and discourage foreign direct investment in GCC countries: Do macroeconomic and environmental factors matter?

**DOI:** 10.1371/journal.pone.0298129

**Published:** 2024-02-15

**Authors:** Majed Alharthi, Md Mazharul Islam, Hawazen Alamoudi, Md Wahid Murad

**Affiliations:** 1 Finance Department, College of Business, King Abdulaziz University, Rabigh, Saudi Arabia; 2 Marketing Department, College of Business, King Abdulaziz University, Rabigh, Saudi Arabia; 3 UniSA Education Futures, University of South Australia, Adelaide, South Australia, Australia; Lahore School of Economics, PAKISTAN

## Abstract

In general, foreign direct investments (FDIs) play a crucial role in driving a country’s economic development, promoting diversification, and enhancing competitiveness. The Gulf Cooperation Council (GCC) countries, which heavily rely on the oil and gas sectors, are particularly vulnerable to fluctuations in commodity prices. However, these countries have recognized the imperative of economic diversification and have increasingly turned to inward FDIs to achieve it. By attracting capital, advanced technology, and expertise from foreign investors, FDIs enable the GCC countries to expand their economic base beyond the oil and gas sectors. This diversification not only creates employment opportunities but also fosters resilient economic growth, ultimately leading to an improvement in the living standards of the local population. This study investigates the macroeconomic and environmental factors that potentially attract foreign direct investment (FDI) inflows into the Gulf Cooperation Council (GCC) countries in the long run. Additionally, the study explores the causal relationship between these factors and FDI inflows. The panel autoregressive distributed lag (ARDL) approach to co-integration is the primary analytical technique used, utilizing long time-series data from six GCC countries, including Bahrain, Kuwait, Oman, Qatar, Saudi Arabia, and the United Arab Emirates (UAE) during the period 1990–2019. The empirical results indicate that, in the long run, almost all independent variables significantly influence FDI in GCC countries. Variables such as GDP growth (GDPG), inflation (INFL), carbon dioxide emissions (CO_2_), and urbanization (URB) are found to be highly significant (p≤0.01) in their impact on FDI. Moreover, unemployment (UNEMP) also positively and significantly influences FDI in these countries in the long run. Based on the key findings, strategies aimed at reducing persistently high unemployment rates, maintaining population growth, viewing FDI as a driver for GDP growth, and continuing with infrastructure development and urbanization are expected to attract more FDI inflows into GCC countries in the long run. Additionally, fostering both long-term economic incentives and creating a conducive business infrastructure for investors are vital for attracting inward FDI into any nation, including those in the GCC. This research would benefit various stakeholders, including governments, local businesses, investors, academia, and the local society, by providing valuable knowledge and informing decision-making processes related to economic development, diversification, and investment promotion.

## 1. Introduction

Foreign direct investment (FDI) is widely acknowledged as a pivotal driver of sustainable economic growth due to its potential to foster innovation, introduce advanced technology, and alleviate poverty [1]. It offers numerous benefits that promote resilient economic development, including the reduction of emissions through technological advancements and investments in renewable energy [1], poverty reduction through job creation and increased tax revenue [2], enhanced production and productivity [3], and transformative effects on a nation’s socioeconomic landscape [4]. Consequently, policymakers and experts view FDI as a vital catalyst for local communities and overall economic improvements, leading to the enactment of laws and policies aimed at attracting foreign investments. Consequently, numerous researchers have dedicated significant efforts to uncovering the factors that either attract or deter FDI over the past few decades.

Numerous studies have explored the determinants of FDI using various methodologies, factors, samples, and timeframes [5–10]. Most of these studies have focused on factors commonly recognized by existing theories, particularly policy indicators, business-related aspects, market-related factors, resource-driven factors, and drivers of economic productivity [9]. Institutional and macroeconomic variables such as taxes, gross domestic product (GDP) growth, inflation, GDP per capita, economic openness, and real exchange rates have been frequently employed in previous studies. Due to the diverse characteristics of both micro and macro environments across different countries and regions, the previously identified determinants of FDI vary significantly from one place to another [11, 12]. For example, [5] conducted a study encompassing 50 developing countries to analyze the determinants of FDI. Their findings highlighted that GDP growth, institutional quality, and human capital played a substantial role in influencing FDI inflows in the sample countries. In a separate study by [9], the authors focused on FDI direction in the fast-growing BRICS (Brazil, Russia, India, China, and South Africa) and MINT (Mexico, Indonesia, Nigeria, and Turkey) countries. They underscored that market size, infrastructure availability, and trade openness were the primary factors attracting FDI to these regions. Nevertheless, all these studies indicated that factors such as the availability of natural resources, political stability, corruption control, inflation rates, and measures of governance had a negative impact on FDI inflows. Also, [6–8] found a positive and significant relationship between FDI and population growth, while [64] discovered the opposite relationship.

The existing empirical evidence, therefore, presents mixed results regarding the determinants of FDI, without a clear consensus on the "true determinants" of FDI [11]. Several factors may contribute to this disparity in empirical findings. Consequently, despite growing interest, the factors influencing FDI remain a subject of ongoing debate [13]. Understanding these determinants holds significant importance for any country seeking to comprehend the forces that shape the flow of FDI into its economy. The findings from such investigations enable governments to formulate appropriate macroeconomic policies aimed at improving competitiveness and making their nation more attractive to foreign investors. However, limited information is available concerning the impact of environmental factors, such as rainfall and temperature, on FDI at the macro level [14, 15]. Thus, it became a popular topic of what the environmental factors of inward FDI are and how they attract and discourage inward FDI under such a complex and uncertain environment.

Gulf Cooperation Council (GCC) countries, including Bahrain, Kuwait, Oman, Qatar, Saudi Arabia, and the United Arab Emirates, are prominent global oil producers and exporters, and their GDPs are heavily reliant on oil exporting revenues, setting them apart from developed and emerging economies [16]. Elevated oil prices stimulate robust economic growth, prompting expansionary strategies, while declining oil prices necessitate contractionary measures like tax increases and government spending cuts. To mitigate this vulnerability, GCC nations have committed to economic diversification, with Saudi Arabia initiating its Saudi Vision 2030 in 2016, which was subsequently followed by other GCC countries. A central objective of Vision 2030 is to reduce oil dependence, given the volatile nature of oil prices. In this context, FDI plays a crucial role in financing development, given its increasing inflow and the limited capacity for local resource mobilization in these nations to meet their growing requirements. Despite their natural resource wealth, trade openness, and affluence, the GCCs have struggled to attract sufficient FDI, raising concerns about their overall progress [17]. This situation suggests that they have yet to create a conducive economic environment to attract greater FDI inflows, particularly considering the vital role FDI plays in sustaining GDP growth in these oil-dependent economies. Consequently, it is imperative to scrutinize the factors impacting FDI, including GDP, inflation, unemployment, demographic metrics, and climatic variables such as CO2 emissions, rainfall, and temperature. Considering the above, extensive literature review, and to the best of our knowledge, there is a significant gap in holistically analyzing the influence of economic, demographic, and climate factors on FDI inflows in the context of GCC countries, which are characterized by deep-rooted cultural and religious traditions. Therefore, this study is an attempt to address the existing research gap by investigating the factors affecting FDI inflows in GCC countries using the panel co-integration technique and time-series data from 1990 to 2019. This study explores the cointegrating relationship between economic, demographic, and climate factors and FDI, contributes much-needed contemporary evidence to this underexplored topic, and addresses the following research questions:

1. What are the significant determinants that attract FDI inflows into the GCC countries?
2. What are the significant and negative determinants of FDI in the GCC countries?
3. Do environmental aspects (such as rainfall and temperatures) affect FDI in the GCC countries?

The study makes two significant contributions. Firstly, much of the prior research on FDI determinants has primarily focused on macroeconomic and demographic factors. This study innovatively integrates climate-related variables into the analytical framework, offering a more holistic examination of the factors influencing FDI. Additionally, it adopts a sustainability perspective by incorporating CO_2_ emissions as an independent variable, positing that nations with lower CO_2_ emissions (indicative of cleaner, more sustainable environments) are likely to emerge as highly appealing investment destinations.

Secondly, this study significantly enriches our comprehension of the GCC countries, a region of paramount global geopolitical and economic importance. Despite their substantial wealth accumulation and distinctive economic model compared to other resource-rich nations, these economies have received limited attention in empirical research. This pragmatic inquiry into the determinants of FDI attraction or deterrence in these nations offers invaluable insights and effectively bridges this research gap. In doing so, it serves as a pioneering investigation that explores a previously unexplored research dimension, making a noteworthy contribution to our understanding of this vital region.

The remainder of this paper is organized as follows: Section 2 presents the study background, focusing on the determinants of attracting and discouraging FDI. Additionally, this section develops the proposed hypotheses of the study. Section 3 provides an outline of the study methodology. Section 4 presents a comprehensive analysis of the empirical data and highlights key results. In Section 5, the vital findings are discussed. Section 6 presents key recommendations. Finally, the paper concludes based on the important results.

## 2. Literature review and hypotheses formulation

### 2.1 Literature review

Historically, [18] sought to define a growth model into a simple production function and to study important factors that may give constant growth rates. In his model, he incorporates factors that influence FDI growth rates. In contrast, according to endogenous growth theory, FDI flows may contribute to an economy’s economic development either directly or indirectly. [19] proposed a two-sector model of international capital flows in which capital flows were seen as a replacement for international commerce, resulting in factor price equality across nations. [19] expanded the theory of comparative advantage by proposing a model that included two nations, two products, two production variables, and two identical production functions in both countries. Mundell’s model, on the other hand, examined more short-term, international portfolio type investments rather than FDI, and hence could not explain worldwide production via FDI. Many of the previous beliefs were centered mostly on the United States and Europe. To address Mundell’s model’s inadequacies, [20] contextualized their model in Japan and advanced the argument that FDI happens when a country has a comparative disadvantage in manufacturing one commodity, but international commerce is dependent on comparative advantage. [21] distinguishes the results of FDI activities into direct beneficial home-country effects, such as increased production and knowledge transfer to domestic suppliers, and indirect effects, such as improved worker quality. In fact, FDI inflows have contributed to EU economic growth since foreign affiliates have a stronger inclination to spend on research and development (R&D) and are more productive when investing in the EU than in their home market [22]. Moreover, the gravity model, which was first used to describe bilateral trade flows between nations using Newton’s law of motion as an analogy [23]. According to the fundamental gravity model, international trade between two countries is determined by the size of their economies as measured by GDP and population, the geographical distance between the two countries, and certain preferential trade factors.

[24] defined FDI “as the amount invested by residents of a country in a foreign company over which they have effective control”. [25] described FDI as “FDI facilitates growth of recipient country via capital formation channels directly and via positive spillovers and inclusion into international productive and innovate networks indirectly”. According to the relevant theories the correlation between FDI and environmental factors can be explained in four hypotheses, including the Porter hypothesis, the pollution haven hypothesis, the Race-to-Bottom hypothesis, and the pollution halo hypothesis. According to the Porter hypothesis, FDI brings new and innovative technologies and may enhance the environmental quality of host countries [26]. On the pollution haven hypothesis, [27] argus that pollution-intensive manufacturing units move from more stringently regulated counties to less stringently regulated countries to reduce the expense of complying with these restrictions. [28] argue that the flight of polluted sectors through FDI to developing countries gives more opportunities that can grow economy but creates environmental concerns in the long-term. [29] argues that the Race-to-Bottom hypothesis and the pollution halo hypothesis explain that FDI in developing countries enhances economic growth and delivers green and innovative technology, hence improving environmental quality. In theory, FDI is thought to influence economic growth primarily through capital accumulation, as well as through introducing current technology and new processes to the host country. To empirically evaluate these theoretical assumptions, the neoclassical and endogenous growth models have been applied on occasion, with variable findings, which might be attributed to different estimates, methodologies, sample sizes, and time periods utilized for this type of research. The literature on FDI has three main topics at microeconomic, macroeconomic, and strategic levels. Our study focuses on macroeconomic levels, which include economic indicators (like GDP, inflation, and unemployment) demographic measures (e.g., population) and climatic issues (CO_2_ emissions, rainfalls, and temperature).

Previous studies have demonstrated the importance of inward FDI for economic growth (e.g., [7]) and job creation (e.g., [30]). However, the literature review revealed that few studies have examined the drivers of inward FDI in GCC countries [31, 32] specifically. This study attempts to fill this gap by examining the main determinants of inward FDI for the GCC countries. However, the GCC countries play an important role in exporting energy resources worldwide. Consequently, studying the determinants of inward FDI is crucial for foreign investors who would like to extend their investments in the GCC region. One of the most recent studies on FDI is Sookram et al.’s (2022) work on Caribbean countries during the period of 2000–2019, which used the Autoregressive Distributed Lag (ARDL) technique to derive its findings. Their results indicate that the correlation between economic development and FDI is significant and positive, and that population growth and the total natural resource rents effectively attract FDI. Surprisingly, over the period of the Global Financial Crisis (GFC: 2007–2009), more foreign investors came to Caribbean countries to expand their businesses. This means that the economies of Caribbean countries were economically stable over that period.

Another recent study conducted by [33] examines the indicators of FDI for West African regions for the period of 1989–2018. In this study, fixed effect model (FEM) and random effect model (REM) were used to estimate the findings. [34] analyze the determinants of FDI in OECD countries during the period of 1990–2020, employing FEM, REM, and generalized method of moments (GMM) regressions. The outcome of the study shows that FDI can be positively driven by economic growth, human capital, and trade openness with other countries. In contrast, the relationship between FDI and physical capital was found to be significant and negative. Surprisingly, the study also indicates that FDI in OECD countries has not been affected by the COVID-19 pandemic. This could be attributed to the infrastructure of investments in OECD countries that are strong and resilient.

Focusing on Saudi Arabia, [35] explore the determinants of inward FDI for the period of 1984–2018, utilizing ARDL approach to cointegration. Their findings show that during the GFC, inward FDI increased significantly in Saudi Arabia due to the high stability of the Saudi economy over this period. In addition, higher rates of institutional quality indices encouraged investors to make FDI into the country. Moreover, Saudi Arabia joined the World Trade Organization (WTO) in 2006, which improved its credibility and trustworthiness in the eyes of investors. Trade openness between Saudi Arabia and other countries also attracted significantly more inward FDI. This study has not investigated the relationship between inward FDI and important factors such as economic growth and inflation, however, we are addressing this gap in our study on the GCC countries. Another study that compares FDI determinants between MENA countries and sub-Saharan countries is [36] for the period of 2000–2012 using the finite element method (FEM). This study also found that higher income per capita and better control of corruption effectively increase FDI, but that higher inflation rates discourage investment in MENA and sub-Saharan countries.

[32] examines the determinants of FDI in the GCC countries over the period of 1980–2013. This study employs three different techniques of FEM, random effect model (REM), and generalized method of moments (GMM) to obtain the findings. The results report that economic growth and labor force have positive effects on FDI. On the other hand, political instability significantly discourages FDI, and higher CPI rates decrease FDI significantly. Furthermore, the relationship between FDI and oil prices was found to be significant and negative. This means that low prices of oil decreased the cost of production and logistics, which encouraged FDI in the GCC countries. Another study on FDI in the GCC countries is [31] work, which looked at the period of 1990–2015, utilizing the techniques of FEM and REM. The results indicate that higher inflation rates significantly increase FDI, and that trade openness supports FDI. The number of mobile subscribers also attracted FDI. The study also found that higher oil prices encourage FDI inflows while higher oil reserves drive FDI to be significantly reduced.

A recent study conducted by [37] on China estimating the factors of FDI over the period 2003–2019. This study employed FEM and REM to analyze the data. The findings reveal that FDI levels increased through higher individual income (GDP per capita). In addition, trade openness has a positive impact on FDI as foreign investors prefer to invest more over the periods of increasing imports from and exports to China. The correlation between FDI and tax revenue is significant and positive as they found. Furthermore, the higher number of population and residents supports FDI rates significantly. On the contrary, they found the association between FDI and financial development significant and negative. Another recent study on China by [38] evaluating the triangle-relationship of industrial pollution, FDI, and economic growth. This study analyzes data obtained from 30 Chinese provinces during the period 2006–2017 using GMM regression. The results of GMM show that the pollution from industrial sulfur dioxide emissions decreased FDI significantly. This result encourages policymakers in the Chinese government to be strict in drawing more policies to decrease pollution which results attracting more FDI and supporting Chinese economy. Another important finding of this study indicates that more investment in environmental pollution control enhances the levels of FDI.

[39] investigate the determinants of FDI of ASEAN+3 countries (China, South Korea, and Japan) through the period 1995–2019 utilizing ARDL models as pooled means group (PMG), means group (MG) and dynamic fixed effects (DFE). According to the results of the long run ARDL-PMG model, more infrastructure of mobile cellular subscriptions led to higher FDI. Moreover, CO_2_ emissions increased with FDI levels significantly, and hence decreased the quality of environment at the same time. In contrast, a negative and significant correlation was found between corruption (measured by the corruption perception index) and FDI, which means that higher levels of corruption discourage foreign investors from expanding their businesses. Outcomes from the long run ARDL-DFE model propose that market size (GDP), infrastructure of mobile cellular subscriptions and corruption increase FDI significantly. On the other side, trade openness impact FDI significantly and negatively. Finally, the results from the long run ARDL-PMG model show that FDI and inflation have a significant but negative relationship.

Focusing on MENA region, another recent study conducted by [40] on five MENA countries (Algeria, Bahrain, Egypt, Syria, and Tunisia). This study examines the indicators of FDI over the period 1980–2014 using OLS, FEM and REM. This study points out that FDI is impacted significantly and positively by economic growth (real GDP). On the other hand, exchange rate volatility, trade openness and political instability affected FDI significantly and negatively. [41] evaluate the variables that affect FDI in 172 countries for the period 2003–2019 employing GMM regression technique. The findings of GMM reveal that a higher number of branches for commercial banks results in having more FDI levels. Also, the government consumption level supports FDI significantly as more governmental spending increases FDI. In addition, the correlation between population density (calculated as mid-year population divided by land area in square kilometers) and FDI is found significant and positive. In contrast, any types of sanctions on any country affected FDI rates significantly and negatively. [42] explore the measures of FDI for 124 counties using the period 1997–2015. The study utilizes baseline and GMM regressions and suggests that real per capita income, GDP growth, urbanization and tax revenue are positive determinants of FDI. While share of shadow economy and trade openness are negative factors for FDI.

[43] study focuses on finding the FDI’s indicators in BRICS (Brazil, Russia, India, China, and South Africa) members through the period 1990–2018. The feasible generalized least squares (FGLS) and dynamic OLS regressions were employed in this study. This study reports that the usage of renewable and non-renewable energy significantly increased FDI in BRICS countries. In addition, GDP and trade openness attract more inflow of foreign capitals to BRICS countries. Finally, higher inflation rates discourage foreign direct investors from investing in BRICS countries over the period of the study.

The study of [44] explores the effects of macroeconomic and environmental variables on FDI for 120 countries for the period 2000–2014. The study utilized OLS and fixed-effects regressions to analyze the data. The outcome of this study reveals that some macroeconomic variables (GDP per capita, trade openness, and real GDP growth) have significant and positive relationships with FDI. Moreover, higher secondary school educational attainment ratio increased FDI significantly. Regarding environmental factors, environmental performance has a significant and positive association with FDI.

[45] examine the correlation between FDI and its determinants for 18 countries over the period 1970–2016. This study used feasible generalized least squares (FGLS) regression to find the significant factors of FDI. The FGLS results point that terrorist attack and political globalization have a significant but negative impact on FDI. Oppositely, macroeconomic factors such as exchange rates, economic growth and trade openness attract more foreign investors. Moreover, social, and economic globalization support FDI significantly.

### 2.2 Hypotheses formulation

Based on the careful searching of the literature and identifying the research gaps, we observe that there is a potential relationship between FDI and the macroeconomic factors of GDP, inflation, unemployment, CO_2_, population, rainfall, temperature, and urbanization. Henceforth, some testable hypotheses, as guided by strong literature support, have been formulated to achieve the key research objectives.

#### 2.2.1 Foreign direct investment and gross domestic product

GDP represents economic growth, so countries with higher GDP representing better economic growth, which makes it an important indicator for countries to stabilize the production of products and services. In addition, healthy GDP allows countries to reduce and diversify risks and better prepares them to face liabilities. Therefore, foreign investors prefer to operate their businesses in countries with higher and stable GDP. Most of the previous studies confirmed that the relationship between GDP growth and FDI is significant and positive. In recent times, many studies investigated it and found a positive relationship between GDP growth and FDI, and they include studies by [40] on MENA countires, [34] on highly emerging BRICS countries, [37] on China, [42] on 124 countires, [1] on Europe, [33] on West African regions, [46] on OECD countries, [6] on Brazil, [47] on China, [7] on Caribbean countries, [48] on China, [49] on 15 countries, [50] on 189 countries, [51] on Greece, [52] on 151 countries, and [53] on China and ASEAN countries. On the other hand, a few studies noted that higher GDP levels reduce FDI significantly, and they include studies by [54] on BRIC countries (Brazil, Russia, India and China) and CIVETS (Colombia, Indonesia, Vietnam, Egypt, Turkey and South Africa) countries, [55] on G20 countries, and [56] on 33 countries. Also, some studies found an insignificant relationship between FDI and GDP growth, and they include [57] on 165 countries, [58] on GCC countries, and [59] on Mexico. Therefore, the first null hypothesis of this study is formulated as:

*H1*: *There is a positive and significant relationship between GDP and FDI in the GCC countries*.

#### 2.2.2 Foreign direct investment and inflation

Theoretically, foreign investors are looking for lower costs of operation and production. This means that lower rates of inflation would attract more foreign investors. In addition, minimizing expenses would maximize profits effectively. However, in the literature review, we can see three different results regarding this relationship. Some studies observed a significant and positive relationship between FDI and CPI indices [31, 55, 60–62]. [60] conducted a study analyzing the factors affecting FDI in Bangladesh for the period 1975–2015. This study points out that foreign investors prefer to invest more when inflation rates are higher. Another study by [61] on India over the period 2009–2017 utilizing FEM shows that the relationship between FDI and inflation rates is significant and positive. Other studies show that higher inflation rates attract more FDIs [43, 54, 56, 63]. [43], for example, examine the FDI determinants of BRICS countries during the period 1990–2018, and they found that higher price indices discourage the levels of FDI significantly. Also, [63] conducted a study on six African countries over the period 1990–2014 employing FEM. The findings of FEM show that there is a significant and negative relationship between FDI and CPI in Africa. However, the results from [64] study reveal that the relationship between inflation levels and FDI is insignificant. [40] have also found similar results in the case of MENA countries. Therefore, the second null hypothesis is formulated as:

*H2*: *There is a positive and significant relationship between FDI and inflation in the GCC countries*.

#### 2.2.3 Foreign direct investment and unemployment

The unemployment rate represents the number of jobless people in a country’s workforce. Higher rates of unemployment increase the rates of deflation due to lower purchasing power, which could affect the inward investments effectively. In fact, the relationship between FDI levels and unemployment rates could be claimed based on the theory that countries with higher rates of unemployment could have lower rates of economic growth. This could reduce foreign investments sharply and significantly. Available literature suggests that higher unemployment rates result in lower intensity of FDI in host countries [30, 65–67]. For example, [67] investigated the main determinants of FDI in 16 African countries for the period 1991–2014, and they found that higher employment rates discourage FDI significantly. Another study by [30] analyzes the drivers of FDI for 15 EU countries for the period 1998–2008 using FEM. The finding of this study reveals that the relationship between FDI and unemployment rates is negative and significant. Some studies, on the other hand, confirmed that foreign direct investors are interested in investing in countries with higher unemployment rates due to low labor costs [67, 68]. The study of [68] investigated the FDI determinants in eight MENA countries for the period 2003–2009 using the technique of Tobit regression. The result of this study indicates that higher rates of unemployment increase the FDI in the MENA region. Some studies, however, found insignificant relationship between FDI and unemployment rates, and they include [69] on Southern African Development Community for the period 1994–2017, [59] on Mexico over the period of 2001–2010, and [70] on 46 African countries for the period 1980–2010. Therefore, the third null hypothesis can be formulated as:

*H3*: *There is a negative and significant relationship between FDI and unemployment in the GCC countries*.

#### 2.2.4 Foreign direct investment and carbon dioxide emissions

The level of carbon dioxide (CO_2_) emissions is crucial for the regulation of climate change and global warming. In most recent studies, the empirical results reveal that levels of FDI increase CO_2_ emissions significantly [39, 49, 71–74]. This shows that higher processes of production for products and services require more non-renewable energy sources, which is why foreign direct investors prefer to invest in countries with higher rates of production and CO_2_ emissions. The study of [49] on 15 countries over the period of 1990–2013 also reveals that higher levels of FDI increase pollution (CO_2_) significantly. Another study by [71] on 21 countries over the period 2003–2013 concludes that the relationship between FDI and CO_2_ emissions is significant and positive in the long run. Also, the study of [72] reports that higher rates of CO_2_ emissions were deemed attractive to the foreign investors in 17 Asian countries during the period 1980–2014. Also, using panel data of 30 provinces in China from 2005 to 2016, [75] found that FDI has a significant positive effect on CO_2_ emission intensity. Furthermore, using a panel dataset of 26 developing countries for the 2011–2021 period, [76] found an interesting result, revealing that FinTech development/investment discourages carbon emissions. In contrast, however, some studies proved the opposite result, finding a negative but significant relationship between FDI and CO_2_ emissions [77–80]. [77], for example, investigated to find the determinants of FDI for 123 countries during the period 1996–2018, and they found that the impact of FDI on CO_2_ is negative but significant. Also, [78] concluded similar results in China for the period 2000–2018. Thus, the fourth null hypothesis is formulated as:

*H4*: *There is a negative and significant relationship between FDI and CO*_*2*_ *in the GCC countries*.

#### 2.2.5 Foreign direct investment and population

The world population is growing rapidly, which can be risky as the resources used to produce goods and services to meet the needs of growing population are limited. However, if the population produces more products and services efficiently, then this can be an opportunity for more investment. Theoretically, the relationship between population growth and FDI could be positive due to the availability of labor force in countries with higher population growth. Numerous studies reported that the relationship between FDI and population growth is positive and significant [6–8, 81]. [6] conducted a study on Brazil for the period 2010–2016, and they pointed that the relationship between FDI and population growth in Brazil is significant and positive. Similarly, [7] examined the correlation between FDI and population growth in the Caribbean countries over the period 2000–2019 utilizing the ARDL approach. The results of ARDL suggest that population growth influences FDI significantly and positively. Moreover, [8] analyzed the factors of FDI for 23 countries during the period 2006–2015 employing FEM. The findings of FEM indicate that the relationship between FDI and population growth is significant and positive. By the contrary, a limited number of studies reported the opposite result and noted a negative relationship between FDI and population growth [64]. As an example, the study of [64] analyzes the drivers of FDI in 107 countries for the period 1984–2009 using GMM technique. The results of GMM suggest that population growth affects FDI significantly and negatively. The above discussion leads to the formation of the fifth null hypothesis on the relationship between FDI and population growth:

*H5*: *There is a positive and significant relationship between FDI and population in the GCC countries*.

#### 2.2.6 Foreign direct investment and rainfall

The unusual rainfall could discourage investors from investing, especially when physical infrastructure encountering with rain risks is weak, which can affect transportations and production systems significantly. There is very limited literature on the relationship between FDI and rainfall in any country or region contexts. [81] examines the effects of rainfall on FDI for the period 2003–2018 in China, and the results of this study confirm that higher amount of rainfall attracts more foreign investments towards China. Another study by [82] shows an opposite result as it tests the determinants of FDI in Nigeria over the period 1981–2017 utilizing ARDL. The findings of ARDL reveal that the relationship between FDI and rainfall is significant and negative. Since the GCC countries’ rainfall and weather conditions match largely to that of Nigeria’s the sixth null hypothesis on the relationship between FDI and rainfall is formulated as:

*H6*: *There is a negative and significant relationship between FDI and rainfall in the GCC countries*.

#### 2.2.7 Foreign direct investment and temperature

Extreme high and low temperatures can affect foreign direct investments negatively as the conditions of work require moderate temperature. In fact, there is very limited research found, examining the relationship between FDI and temperature even though the temperature is a very important factor influencing foreign direct investment decisions. In an extensive various country-level study, [83] evaluate the influence of temperature on FDI for high income, developing, upper middle income, lower middle income, and low-income countries over the period 1995–2014 employing the ARDL technique. The results of this study suggest that higher temperature levels attract more FDI to high income countries and that more temperature levels decrease FDI significantly in developing and upper middle-income countries. Based on the findings of [83], it is difficult to come up with a single null hypothesis in the context of GCC countries, which share common economic characteristics. One of the most recent studies by [84] examined the connectedness between climate risks and FDI for emerging and advanced countries through the period 2010–2020, but they found an insignificant and positive correlation between temperature and FDI. Since the GCC countries are generally considered high income countries the seventh null hypothesis on the relationship between FDI and temperature is formulated as:

*H7*: *There is a positive and significant relationship between FDI and temperature in the GCC countries*.

#### 2.2.8 Foreign direct investment and urbanization

Urbanization indicates the number of populations that live in urban areas compared to rural areas. Foreign investors prefer to invest in countries with higher proportions of urbanization due to higher income of urban populations and better urban infrastructure compared to rural areas. In one of the previous studies, [85] investigated the relationship between FDI and urbanization in China for the period 2004–2012 using the technique of GMM regression. The result of GMM indicates that foreign investors prefer in investing in China when the urbanization percentage is higher. This result is in line with the finding of [42], who used an extensive panel dataset of 124 nations between 1997 and 2015. By the contrary, the study of [86] on China during the period 2004–2016 found that urbanization percentage impacts FDI levels negatively and significantly. Also, [64] conducted a study on 107 countries over the period of 1984–2009 using GMM and found that the relationship between FDI and urbanization is negative and significant. In contrast to above studies, [87] found a positive but insignificant relationship between FDI and urbanization proportion in Indonesia for the period 2004–2012. Since the GCC countries have been going through rapid urbanization, especially over the last three decades, the eighth but the last null hypothesis is formulated as:

*H8*: *There is a positive and significant relationship between FDI and urbanization in the GCC countries*.

**Fig 1:**
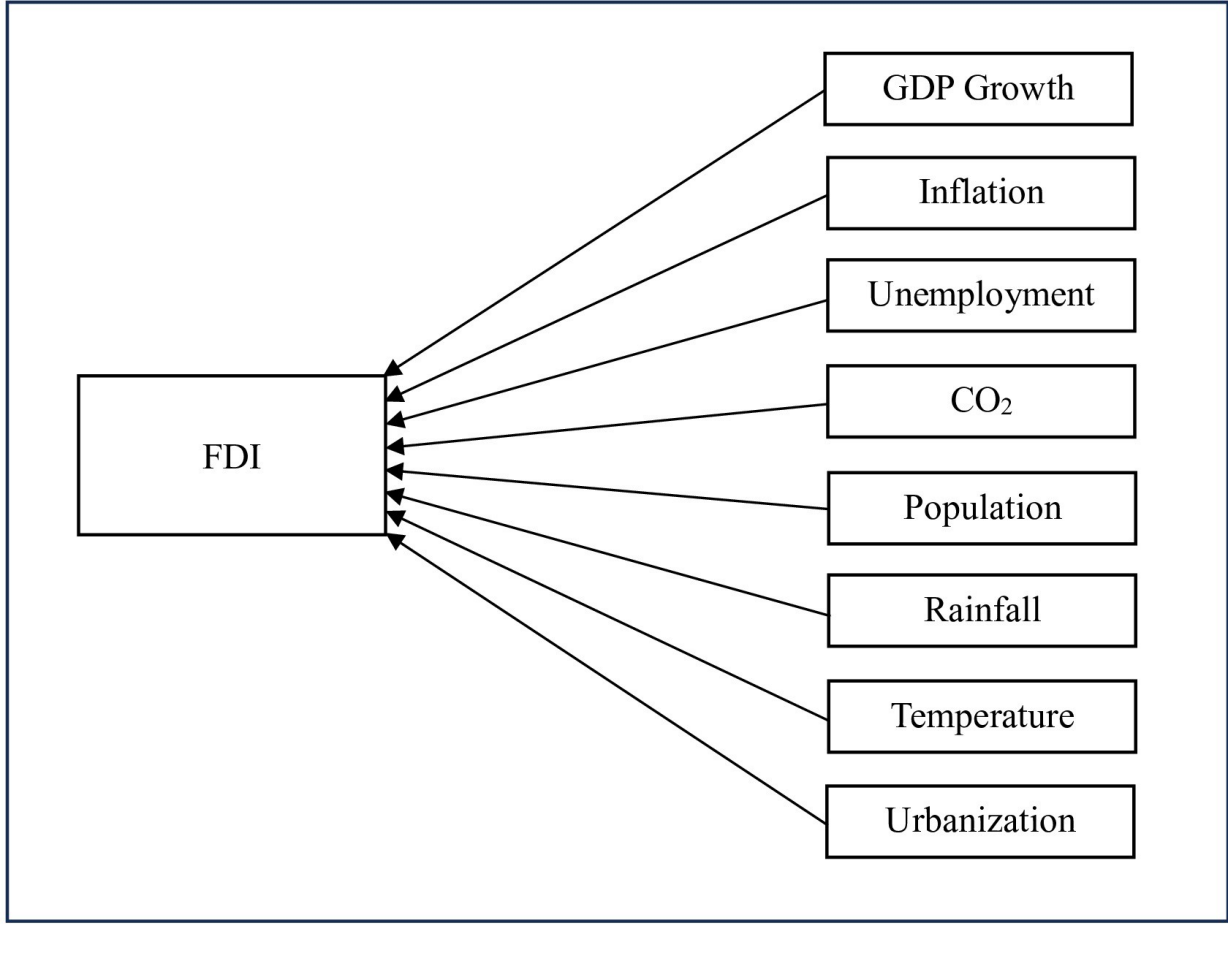
The conceptual framework of the study.

Fig 1 conceptualizes all the above-mentioned hypotheses in respect of FDI inflows into the GCC countries.

## 3. Methodology

### 3.1 Data

The data for this study were collected from two sources, including the World Bank, and the Climate Change Knowledge Portal for Development Practitioners and Policy Makers by World Bank, covering the period from 1990 to 2019. The sample for the study consisted of six countries in the GCC region: Bahrain, Kuwait, Oman, Qatar, Saudi Arabia, and the United Arab Emirates (UAE). The dependent variable in this study is FDI, and the independent variables include GDP, inflation, unemployment, CO_2_ emissions, rainfall, temperature, and urbanization. The study had a total of 180 observations for each variable, which indicates that data from these variables were collected over a span of 30 years and from six countries as mentioned above. Table 1 below includes short definitions of variables and their sources.

**Table 1:** Definitions of variables and sources.

Variable	Definition	Source
FDI	% FDI, net inflows % of GDP	World Bank [88]
GDP Growth	% Gross domestic production growth	World Bank [88]
Unemployment	% Unemployment rate	World Bank [88]
CO_2_	CO_2_ emissions (metric tons per capita)	World Bank [88]
Population	Natural logarithm of total population	World Bank [88]
Rainfall	% Rainfall change	Climate Change Knowledge Portal [89]*
Temperature	Natural logarithm of mean temperature	Climate Change Knowledge Portal [89]*
Urbanization	% Urban population growth	World Bank [88]

* Climate Change Knowledge Portal for Development Practitioners and Policy Makers by World Bank

### 3.2 Variables and model

Since this study utilized time-series data, the main analytical approach applied was the panel ARDL to co-integration technique. The primary investigation focused on examining the relationships between foreign direct investment (FDI) and various variables in the six GCC countries. The variables of interest included gross domestic production growth (GDPG), inflation (INFL), unemployment (UNEMP), carbon dioxide (CO_2_) emissions, population growth (POPL), rainfall (RFL), temperature (TEMP), and urbanization (URB). The study assessed whether these variables, namely GDPG, INFL, UNEMP, CO_2_, POPL, RFL, TEMP, and URB, were integrated into the model as explanatory variables, while FDI served as the dependent variable in the long run. The simplistic econometric model specification can be presented as follows:

$$\text{FDI}=\text{f}\left(\text{GDPG},\text{INFL},\text{UNEMP},{\text{CO}}_{2},\text{POPL},\text{RFL},\text{TEMP},\text{URB}\right)$$

In the above equation, FDI is expressed as a function of GDPG, INFL, UNEMP, CO_2_, POPL, RFL, TEMP, and URB.

If the estimated econometric model above is not in linear form, it would not yield reliable and consistent results, rendering them less useful for decision-making [90, 91]. To address this concern, all variables were converted into their natural logarithms to examine the relationships between the independent and dependent variables. The primary rationale for using a log-linear specification model is to achieve better, more consistent, and reliable empirical results [92, 93]. The log-linear functional form of the model is presented below:

$$\begin{array}{c} LnFDI_{i,t}=b_{0}+b_{1}*LnGDPG_{i,t}+b_{2}*LnINFL_{i,t}+b_{3}*LnUNEMP_{i,t}+b_{4}*LnCO2_{i,t}+b_{5}\\ {}*{\text{LnPOPL}}_{i,t}+b_{6}*LnRF_{i,t}+b_{7}*LnTEMP_{i,t}+b_{8}*LnURB_{i,t}+c_{i}+\varepsilon_{it} \end{array}$$

where countries are denoted by the subscript i (i = 1,.. ., N); the time period is denoted by the subscript t (t = 1,.. ., T); and b1, …, b4 are the coefficients of the regressors estimated by the regression analysis. These coefficients imply that assuming all other variables remain constant, a one-unit change (+/-) in one explanatory variable would change FDI by b units. Ci represents an unknown country-specific constant (the “fixed effect”), and ε_it_ is the random error term capturing all factors that influence FDI. The above log-linear function has the form of an ARDL(p, q, q…, q) model that can be presented as follows:

$$FDI_{i,t}=\sum_{j=1}^{p}\alpha_{ij}FDI_{i,t-j}+\sum_{j=0}^{q}\delta_{ij}X_{i,t-j}+\mu_{i}+\varepsilon_{it}$$

where X is the vector of explanatory variables; however, when reparametrizing the model, it turns into the following form:

$$FDI_{i,t}=\varphi_{i}\left(FDI_{i,t-1}-\beta_{i}X_{it}\right)+\sum_{j=1}^{p-1}\alpha_{ij}\Delta FDI_{i,t-j}+\sum_{j=0}^{q=1}\delta_{ij}\Delta X_{i,t-j}+\mu_{i}+\varepsilon_{it}$$

## 4. Empirical findings

### 4.1 Descriptive statistics

Prior to applying the ARDL analytical approach to cointegration and developing an ARDL model to understand the long-run relationships among the time-series variables, it was essential to examine the descriptive statistics. This step allowed for the assessment of data normality and adequacy through the observation of various statistics (Table 2). Among all the variables, low standard deviations were observed, except for POPL and TEMP, which demonstrated steady variations in their values. On the other hand, the remaining variables showed higher, if not extreme, variations. The mean and median for all variables closely coincided, indicating normality in the data sets. Additionally, except for the four variables of GDPG, CO_2_, RFL, and URB, the skewness for all other variables was found to be within the range of -1 to +1. Furthermore, the probability value for all the variables was statistically significant, implying that the datasets of all the GCC countries were adequate and suitable for further analysis in their present form.

**Table 2:** Descriptive statistics of the variables.

	FDI	GDPG	INFL	UNEMP	CO_2_	POPL	RFL	TEMP	URB
Mean	0.032	0.879	0.591	0.429	0.955	2.699	0.568	1.191	1.310
Median	0.137	1.160	0.765	0.573	1.083	2.687	0.604	1.191	1.483
Maximum	1.969	2.739	4.372	1.445	1.284	2.854	1.046	1.217	1.527
Minimum	-2.298	-2.381	-3.361	-1.533	-1.870	2.571	-1.870	1.157	-1.870
Std. Dev.	1.126	0.889	1.526	0.737	0.355	0.078	0.320	0.014	0.464
Skewness	-0.324	-1.611	-0.075	-0.623	-3.859	0.351	-3.561	-0.089	-3.204
Probability	0.002	0.000	0.016	0.001	0.000	0.036	0.000	0.011	0.000
Observations	180	180	180	180	180	180	180	180	180

### 4.2 Correlation analysis

Correlation analysis was conducted to examine the presence of significant correlations among the variables. Among all 18 correlations, the t-statistical probabilities were found to be significant (Table 3). The correlation coefficients ranged from 0 to 1, indicating no correlation to perfect correlation between the variables. Each variable had the highest correlation coefficient with itself, which was 1, and its correlation with other variables was lower than its own correlation value. Thus, the discriminant validity of the variables was ensured. Notably, eight pairs of correlations generated relatively higher coefficients with higher significance (p ≤ 0.01). These correlations were observed between CO_2_ and UNEMP, POPL and UNEMP, TEMP and POPL, TEMP and UNEMP, URB and UNEMP, URB and CO_2_, URB and POPL, and URB and TEMP. It is essential to note that correlation does not imply causation. However, these findings can be compared with other results, particularly the Granger causality test results presented later.

**Table 3:** Correlation analysis of the variables.

Correlation									
t-Statistic									
Probability	FDI	GDPG	INFL	UNEMP	CO_2_	POPL	RFL	TEMP	URB
FDI	1.000								
	‐‐‐‐								
	‐‐‐‐								
GDPG	0.100	1.000							
	1.343	‐‐‐‐							
	0.181	‐‐‐‐							
INFL	0.062	0.004	1.000						
	0.835	0.055	‐‐‐‐						
	0.405	0.956	‐‐‐‐						
UNEMP	-0.028	0.109	-0.096	1.000					
	-0.374	1.458	-1.286	‐‐‐‐					
	0.709	0.147	0.200	‐‐‐‐					
CO_2_	-0.061	0.079	0.218	0.381	1.000				
	-0.810	1.050	2.973	5.498	‐‐‐‐				
	0.419	0.295	0.003	0.000	‐‐‐‐				
POPL	-0.122	0.110	-0.058	0.713	0.237	1.000			
	-1.640	1.471	-0.779	13.572	3.253	‐‐‐‐			
	0.103	0.143	0.437	0.000	0.001	‐‐‐‐			
RFL	-0.122	-0.058	-0.119	0.162	0.160	0.090	1.000		
	-1.646	-0.781	-1.601	2.183	2.163	1.205	‐‐‐‐		
	0.102	0.436	0.111	0.030	0.032	0.230	‐‐‐‐		
TEMP	0.333	-0.052	0.289	-0.586	-0.141	-0.508	-0.366	1.000	
	4.710	-0.688	4.030	-9.652	-1.896	-7.863	-5.249	‐‐‐‐	
	0.000	0.492	0.000	0.000	0.060	0.000	0.000	‐‐‐‐	
URB	-0.088	0.147	-0.038	0.591	0.926	0.353	0.244	-0.346	1.000
	-1.175	1.987	-0.506	9.785	32.593	5.033	3.352	-4.926	‐‐‐‐
	0.242	0.048	0.614	0.000	0.000	0.000	0.001	0.000	‐‐‐‐

### 4.3 Unit root test

The stationarity of the dataset was assessed due to its significance in evaluating time-series data, especially in the case of macroeconomic data. To determine whether the data were stationary, two panel unit root tests, the Augmented Dickey Fuller (ADF) test and the Levin, Lin, and Chu (LLC) test, were applied. The ADF test is commonly used to identify serial correlation in time-series analysis and to assess whether a given time-series dataset is stationary. Additionally, the ADF-Chi-squared test, a non-parametric test, was used to nullify the null hypothesis concerning the variables’ relationship. The Levin, Lin, and Chu (LLC) test was employed to assess the level of bias in the null hypothesis, thus enhancing the strength and scope of the panel unit root test. The null hypothesis of the LLC test assumes that the data is not stationary, while the alternative hypothesis suggests that the data set is stationary.

**Fig 2:**
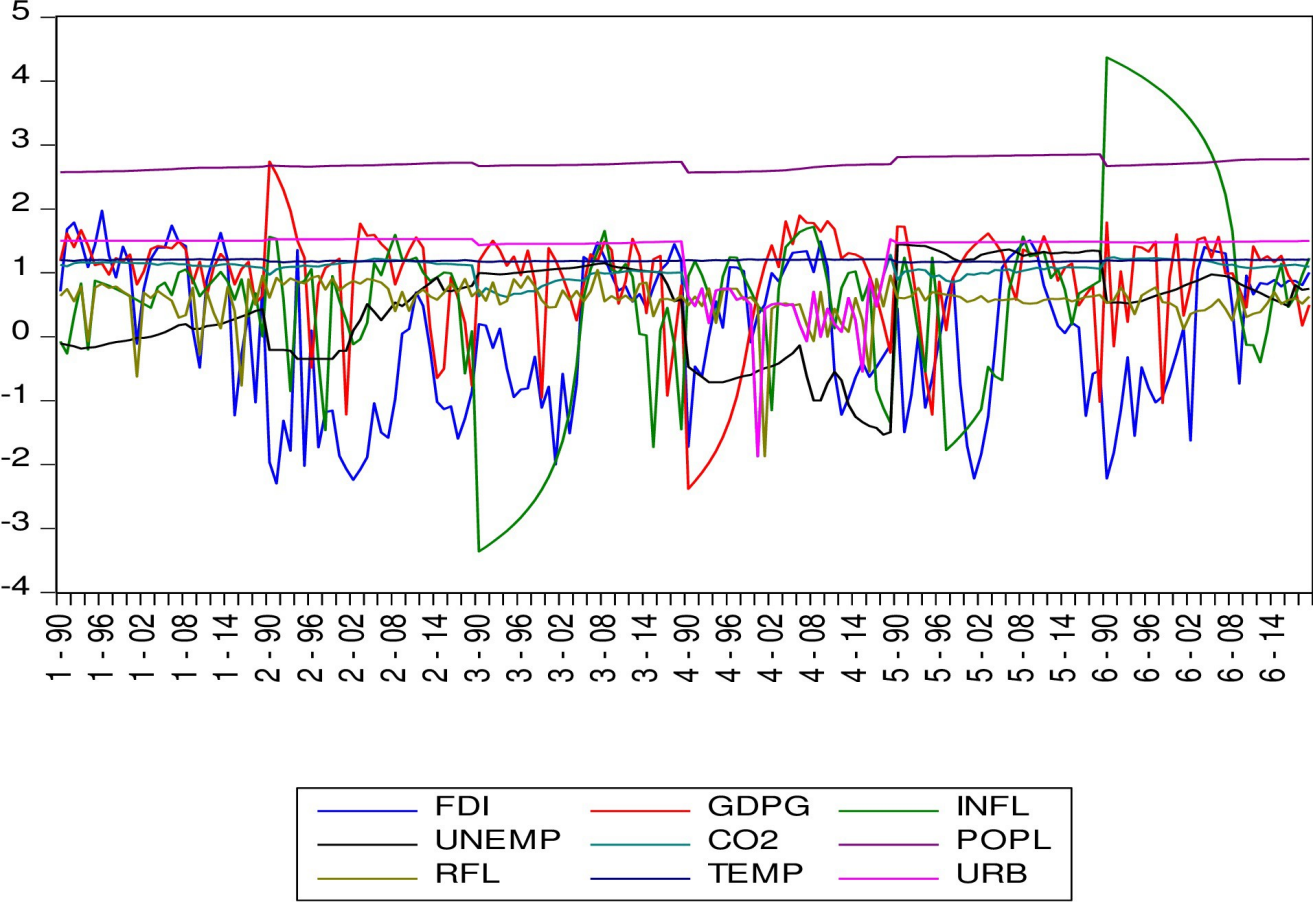
Combined trends of all variables.

**Fig 3:**
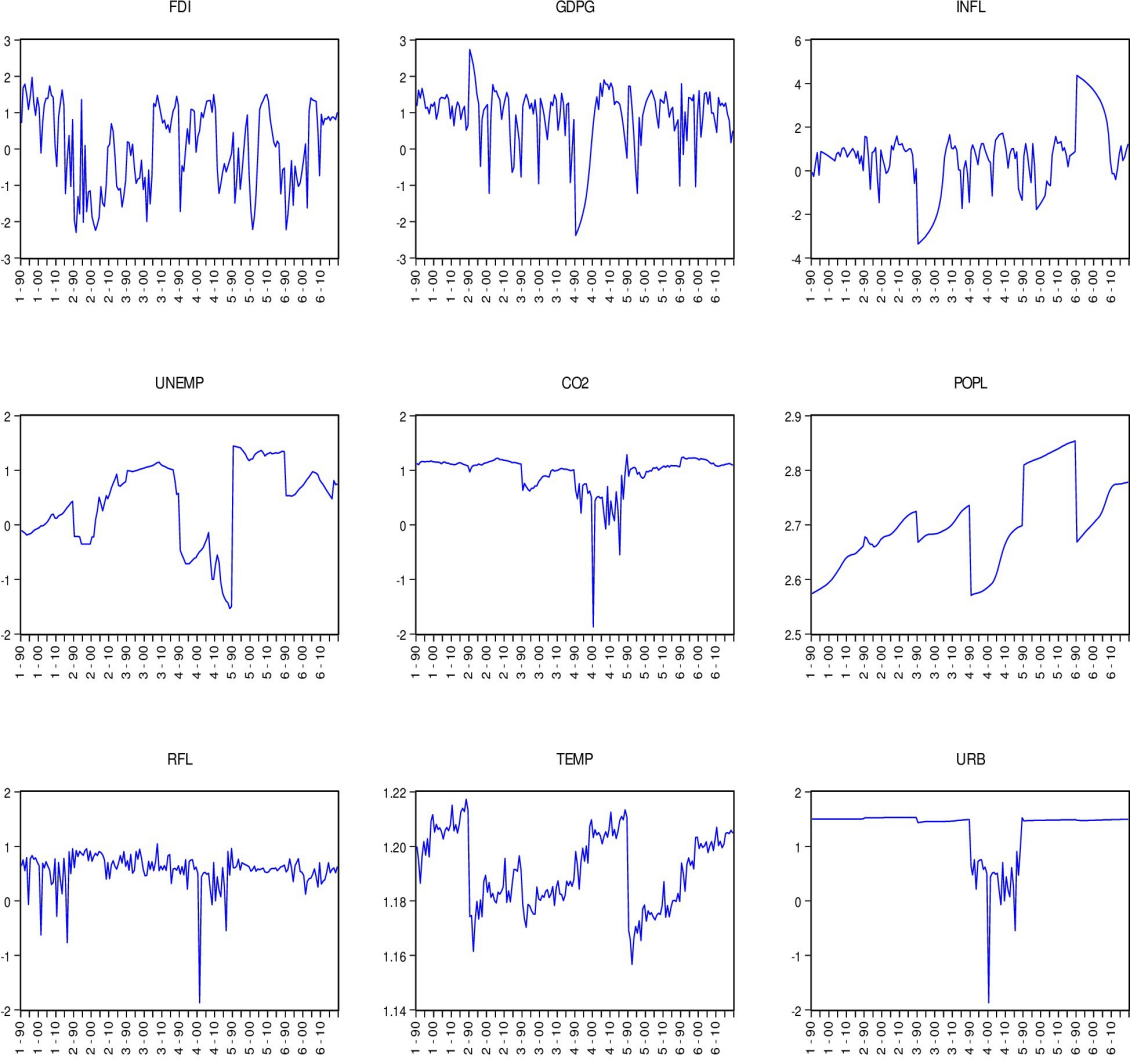
Individual trend of variables.

Upon conducting the ADF and LLC panel unit root tests (Table 4), the results indicated that the variables were statistically significant at either I(0), I(1), or a combination of both, indicating mixed orders of stationarity. It is worth noting that the ARDL approach to co-integration can be applied regardless of whether the underlying variables are I(0), I(1), or a combination of both, but it cannot be applied when the underlying variables are integrated at order I(2). However, the results from the panel unit root test revealed that all the variables became stationary at first difference, leading to the rejection of the null hypothesis. As a result, the conditions for applying the ARDL approach were met for the datasets of all six GCC countries. Fig 2 indicates the combined trends of all variables, that caused a mixed order of integration as confirmed by the unit root tests. Fig 3 shows the Individual trend of variables, which confirm again a mixed order of integration in the long run as confirmed by the unit root tests.

**Table 4:** ADF and LLC panel unit root test results.

Constructs	ADF Test		LLC Test	
	Level	First Diff.	Level	First Diff.
FDI	17.5550	52.4962***	-0.67406	-3.7050***
GDPG	24.9930***	69.8325***	1.1309	-2.8328***
INFL	14.1881	60.0847***	0.5722	-5.9326***
UNEMP	12.0138	53.3121***	-0.5169	-7.0130***
CO2	17.6151	52.3458***	0.1482	-3.4253***
POPL	123.0490***	67.1975***	-10.9640***	-7.1933***
RFL	30.4563***	98.2719***	-0.0554	-2.2783***
TEMP	31.6395***	127.2230***	-3.6653***	-1.4756*
URB	44.8604***	305.6120***	1.6373	-63.0279***

Note: ADF = Augmented Dickey-Fuller test for unit root, LLC = Levin, Lin & Chi test, (*) significant at 10% level; (**) significant at 5% level; (***), significant at 1% level.

### 4.4 Co-integration test

After confirming that the variables were stationary, the next step was to assess whether they were co-integrated. The co-integration test was conducted, and the results are presented in Table 5. This test helps determine the degree of responsiveness between two variables over a specific period and identifies correlations between multiple time-series variables. Four types of statistics were observed in this test, including the statistic, probability, weighted statistic, and probability. The individual AR coefficient and Kao test result were examined against the p-values using the error correction model and adjustment coefficients to calculate the forces influencing the relationship between two variables towards the long-run equilibrium. The primary focus was on the p-value against the "group PP statistic" to determine co-integration between two variables, if not among all of them.

**Table 5:** Co-integration test of variables.

Alternative hypothesis: common AR coef. (within-dimension)				
			Weighted	
	Statistic	Prob.	Statistic	Prob.
Panel v-Statistic	-1.60097	0.9453	-1.6881	0.9543
Panel rho-Statistic	1.469247	0.9291	1.510479	0.9345
Panel PP-Statistic	-2.92043	0.0017	-3.84892	0.0001
Panel ADF-Statistic	0.904343	0.8171	-0.11546	0.454
Alternative hypothesis: individual AR coef. (between-dimension)				
	Statistic	Prob.		
Group rho-Statistic	2.442706	0.9927		
Group PP-Statistic	-5.55513	0.0000		
Group ADF-Statistic	0.450178	0.6737		
Kao Test	Statistic	Prob.		
ADF	-2.238058	0.0126		

The empirical results in Table 5 indicate that the p-values for all these statistics were less than 0.05, leading to the rejection of the null hypothesis. As a result, it was concluded that significant co-integration existed among the variables. This finding highlights the importance of conducting long-run estimations using the data sets, as the relationships among the variables are established over the long run.

### 4.5 Heteroscedasticity test

To assess whether there were any heteroscedasticity issues in the ARDL model, a heteroscedasticity test was conducted. This test is commonly used in the analysis of linear regression models estimated using time-series variables. It specifically examines whether the error terms for all the variables are not the same and tests the null hypothesis concerning the presence of heteroscedasticity in the estimated model. Additionally, it checks whether the variance of the regression errors depends on the values of the independent variables. The null hypothesis in this test assumes that "there is no heteroscedasticity problem in the estimated model," while the alternative hypothesis assumes otherwise, i.e., "there is a homoscedasticity problem in the estimated model." However, the empirical results in Table 6 indicate that the p-value of the heteroscedasticity test was greater than 0.05, leading to the inability to reject the null hypothesis. Therefore, it was concluded that there was no heteroscedasticity problem found in the estimated model, and the error terms for all variables used in the model were not significantly different.

**Table 6:** Heteroscedasticity test results.

Dependent Variable	S. Value	DF	Probability
FDI	6.63345	6	0.3561

### 4.6 CUSUM test

**Fig 4:**
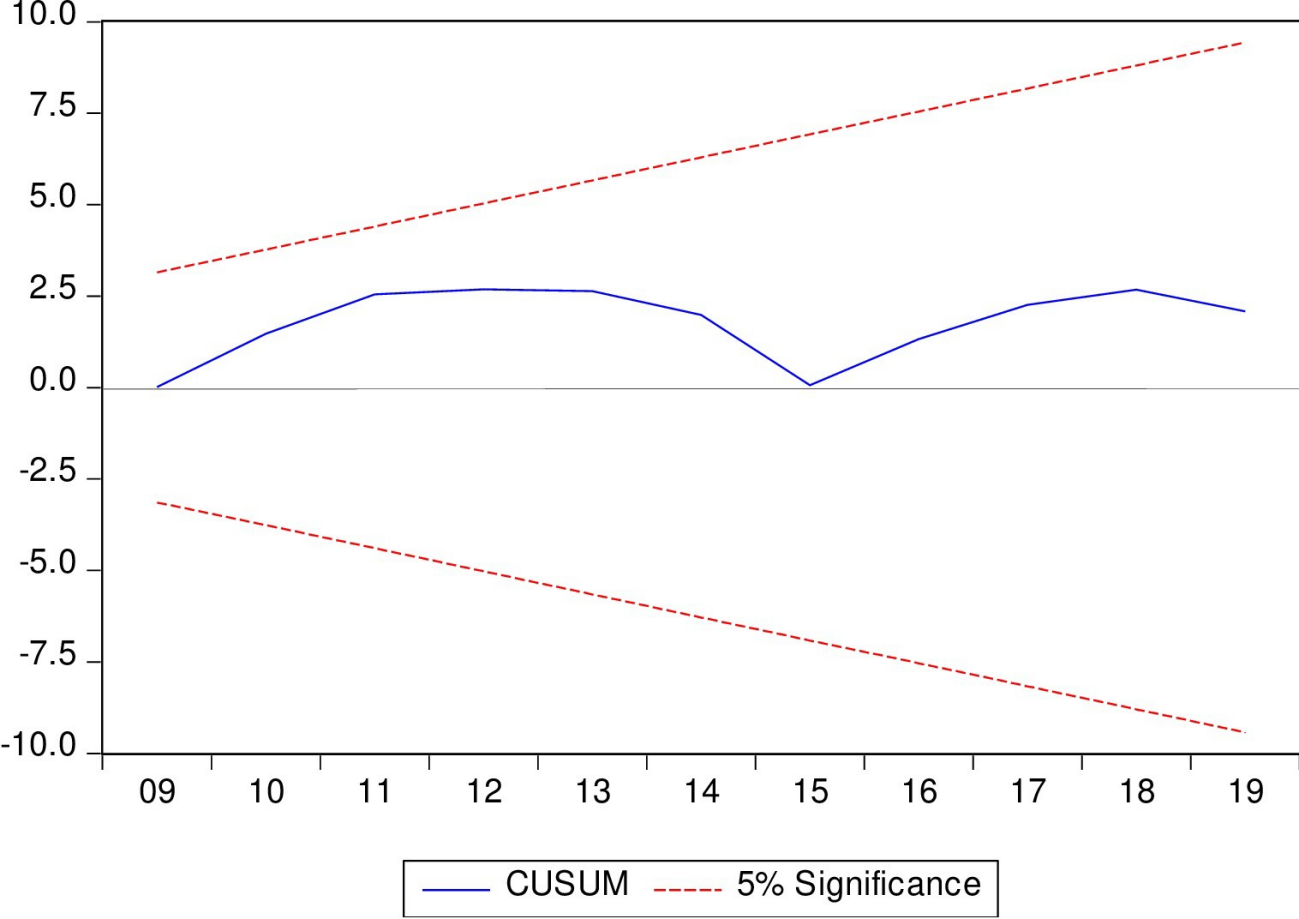
CUSUM test graph.

**Fig 5:**
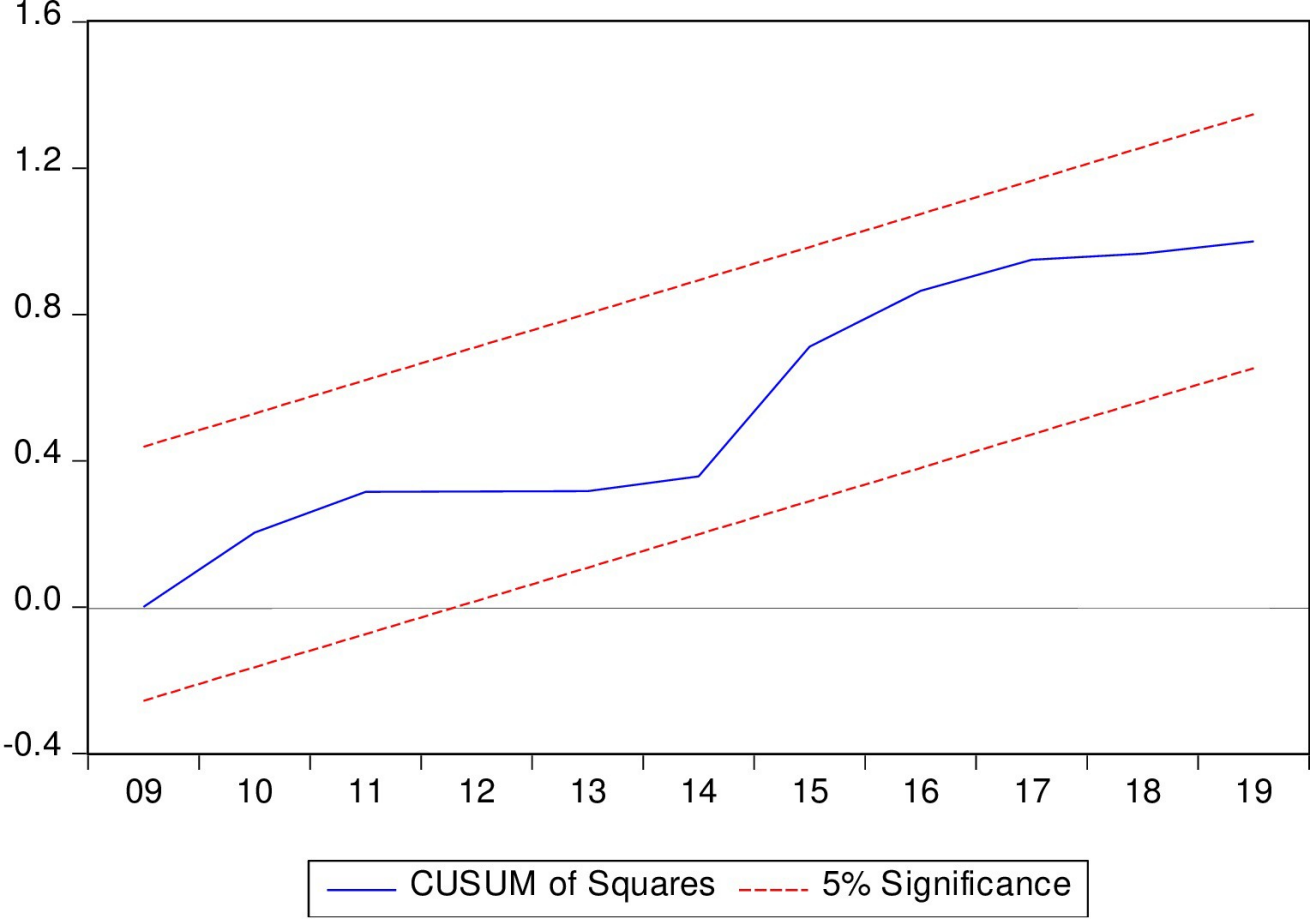
CUSUM squares test graph.

The CUSUM test is utilized to test for instability in the intercept, while the CUSUM of squares is employed to test for instability in the variance of the regression error. In the estimated long-run ARDL model, both aspects have demonstrated stability, further confirming the model’s acceptability. Figs 4 and 5 present plots with constant upper and lower bounds for a 5% significance level, used as a test for parameter stability. In both figures, the CUSUM statistic remains within the upper and lower bounds, leading us to not reject the null hypothesis of parameter stability. These plots also provide additional information on the timing of any structural break.

### 4.7 The ARDL model: Long-run and short-run estimations

As mentioned earlier, the necessary conditions for applying an ARDL model were found to be satisfactory, and thus, the ARDL approach to time-series variables was utilized to estimate both the long-run and short-run coefficients (Table 7). Surprisingly, in the short-run estimation, none of the eight independent variables were found to be statistically significant. This suggests that during the study period, none of the eight independent variables significantly attracted or discouraged FDI in the GCC countries. However, it is noteworthy that three variables, namely POPL, RFL, and TEMP, had a positive influence on FDI in those countries during the same period, albeit not statistically significant. The implications of these positive influences on attracting FDI to those countries will be critically discussed in the next paragraph, along with the theoretical underpinnings of the findings.

**Table 7:** ARDL model estimations in long run and short run.

Variable	GDPG	INFL	UNEMP	CO_2_	POPL	RFL	TEMP	URB
Long Run Estimation	0.518***	0.533***	1.506*	-7.690***	11.516	0.469	2.752	6.963***
	(3.839)	(5.932)	(1.939)	(-3.795)	(0.830)	(1.603)	(0.119)	(2.581)
Short Run Estimation	-0.003	-0.120	-0.691	-2.857	103.818	0.125	3.598	-258.945
	(-0.015)	(-1.328)	(-0.467)	(-0.515)	(0.515)	(0.273)	(0.290)	(-0.907)

In the long run, all but three independent variables have significantly influenced FDI in the GCC countries. Four independent variables, namely GDPG, INFL, CO_2_, and URB, have highly significantly (p ≤ 0.01) influenced FDI, while UNEMP had a positive and somewhat significant influence (p ≤ 0.1) on the dependent variable in the long run. Specifically, a one-unit increase in UNEMP caused FDI to increase by 1.506 units in the long run. This finding might be considered unusual in general, but not for the GCC countries, which heavily rely on a foreign skilled, semi-skilled, and unskilled workforce, while experiencing increasing unemployment among their own work-age populations in the long run. A comprehensive theoretical explanation of this unusual finding is provided in the discussion in the following section. On the other hand, the other three independent variables, namely POPL, RFL, and TEMP, all positively but insignificantly influenced FDI in those countries during the same period.

Among the highly significant independent variables, GDPG, INFL, and URB were found to have a positive influence on FDI, indicating that a one-unit increase in GDPG, INFL, and URB in GCC countries leads to a highly significant long-term increase in FDI by 0.518, 0.533, and 6.963 units, respectively. Therefore, these were identified as the key independent variables that potentially attract or discourage FDI in GCC countries in the long run. However, the independent variable CO_2_ had a negative but highly significant influence on FDI in the long run, suggesting that an increase in CO_2_ in the GCC countries leads to a decrease in FDI in the long run. Specifically, a one-unit increase in CO_2_ caused a highly significant decrease in FDI by 7.690 units.

### 4.8 Granger causality test

The Granger causality test was conducted on all the variables considered in the long-run estimation, and the summary results are presented in Table 8. Examining the overall causality scenario in the table below, it becomes evident that the Granger causality test has generated strong causality statistics for all variables. For instance, the empirical results revealed statistically highly significant (p ≤ 0.01) one-way causalities running from CO_2_ to INFL, POPL to RFL, TEMP to RFL, URB to RFL, RFL to URB, CO_2_ to RFL, and URB to UNEMP. The one-way causalities between the other pairs of variables, as depicted in Table 7, were also significant at other probability levels (p ≤ 0.05). Of significant importance, the causality test did not yield any significant inverse causality; instead, strong causalities were observed from the dependent variable to all the independent variables in this study.

**Table 8:** Granger one-way causality test statistics.

Direction of Causality	Obs	F-Statistic	Prob.	Results
FDI → RFL	168	3.06190	0.055	FDI Granger cause RFL
INFL →TEMP	168	3.12896	0.047	INFL Granger cause TEMP
URB →INFL	168	3.05548	0.050	URB Granger cause INFL
CO2 →INFL	168	4.39315	0.014	CO_2_ Granger cause INFL
POPL→RFL	168	5.06992	0.007	POPL Granger cause RFL
UNEMP →POPL	168	3.01645	0.052	UNEMP Granger cause POPL
TEMP →RFL	168	8.50753	0.000	TEMP Granger cause RFL
URB →RFL	168	62.3587	0.000	URB Granger cause RFL
RFL→URB	168	4.57680	0.012	RFL Granger cause URB
CO2 →RFL	168	63.3511	0.000	CO_2_ Granger cause RFL
RFL→CO2	168	3.41286	0.035	RFL Granger cause CO2
URB →UNEMP	168	4.36558	0.014	URB Granger cause UNEMP
CO2 →UNEMP	168	3.63379	0.029	CO_2_ Granger cause UNEMP
$Co_{2}\underset{➝}{\to }URB$	168	3.57850	0.030	CO_2_ Granger cause URB

## 5. Discussion

In the previous section, we discussed the long-run relationships between FDI and all the independent variables. Now, we compare our empirical findings with those obtained by recent and relevant studies. This comparison can help shed light on the key findings and highlight the theoretical contributions of this study to the existing literature. Firstly, the long-run observation of the relationship between UNEMP and FDI was found to be positive and significant, which aligns with the findings of [68] in the case of selected Middle East and North African (MENA) countries. They argued that the low labor costs resulting from high unemployment rates in these countries attract FDI, which appears to be the case in GCC countries as well. However, a conflicting result was obtained by [59] in the case of Mexico, where they found an insignificant correlation between FDI and unemployment rates over the period 2001–2010.

While several studies [39, 49, 71–75] have found a positive and significant long-run relationship between FDI and CO_2_ emissions, we found a negative but highly significant (p≤0.01) relationship between the two in the case of GCC countries. Such a negative influence of FDI on CO_2_ emissions is in harmony with the findings obtained very recently by [38, 76–80]. Usually, high CO_2_ emissions due to the absence of regulatory carbon emission policy instruments discourage inward FDI, and hence, a negative influence of FDI on CO_2_ emissions is a likely outcome.

While we obtained a positive but insignificant relationship between FDI and population in the case of GCC countries, some recent studies found slightly different results in various contexts. For example, [6–8] found a positive and significant relationship between FDI and population growth in the cases of Brazil, Caribbean countries, and selected emerging countries, respectively. On the other hand, a study conducted by [64] found a negative long-run relationship between FDI and population growth in a large-scale panel study conducted on 107 countries. Therefore, whether population growth attracts FDI remains inconclusive. What is conclusive, however, is that a country’s political, economic, social, technological, environmental, and legal contexts play an important role in determining the relationship between FDI and population growth.

Our empirical finding of a positive and highly significant (p ≤ 0.01) long-run relationship between FDI and inflation in the case of GCC countries is consistent with the results of [30, 31, 55, 60–62]. The phenomenon of higher inflation with higher inward FDI is widely accepted across countries, be they developing, emerging, or developed. The substantial job creation resulting from inward FDI in host countries boosts the income of residents, leading to inflationary pressure on the prices of the goods and services they purchase.

In this study, a positive but insignificant long-run relationship between FDI and rainfall in the case of GCC countries indicates that it has limited policy implication and decision-making significance. While [81] found a positive and significant relationship between FDI and rainfall, the study by [82] found an opposite result. So, the long-run relationship between the two variables should be interpreted with caution. As of now, no studies have found evidence of rainfall leading to FDI or vice versa, so a correlation between the two could somewhat be the outcome in certain circumstances. However, it can be argued that optimal rainfall in any country positively contributes to the local economy and makes the nation attractive for FDI. Therefore, a positive relationship between FDI and rainfall may be of interest to policy and decision-makers within and outside the relevant countries.

Obtaining a positive but insignificant long-run relationship between FDI and temperature in the case of GCC countries sounds interesting but of low policy implication due to the lack of robustness of that relationship. While the investigation by [83] found higher temperature levels attracting more FDI in high income countries, a very recent study by [84] also found a positive but insignificant correlation between FDI and temperature. In fact, the literature on the long-run relationship between these two variables is not extensive enough, however, it is reasonable to argue that a favorable and conducive temperature would attract an inward FDI to countries that are endowed with such favorable conditions.

We discovered a positive and highly significant (p ≤ 0.01) long-run relationship between FDI and GDPG variables, which aligns with recent findings by [7, 46, 52] in the cases of OECD, selected Caribbean, and 43 home and 151 host countries, respectively. Traditionally, FDI contributes to GDPG, so a positive and significant impact of FDI on GDPG is expected. However, one study by [55] found the opposite result in G20 countries, suggesting that higher GDPG reduces FDI significantly. Given that G20 countries are the most economically developed and may no longer be as attractive as the emerging and GCC countries for FDI, such a finding is not surprising.

A positive and highly significant long-run relationship between FDI and INFL is both theoretically and practically acceptable. This study has confirmed this relationship, which aligns with the recent findings obtained by [31, 55, 60] in the cases of Bangladesh, G20 countries, and oil-exporting countries (OPEC), respectively. Other studies by [43, 54, 63], have also observed that higher inflation rates attract more FDIs in BRICS and CIVETS, BRICS, and African countries, respectively. Therefore, the link between FDI and INFL does not warrant further critical discussion.

Using an extensive panel of 107 countries over the period 1984–2009, [64] found a negative but significant long-run relationship between FDI and URB. However, in the case of GCC countries, we found a positive and highly significant relationship between them, which is well supported by [42, 85]. The GCC countries are going through rapid urbanization, which is often associated with economic growth, urban periphery expansion and attractive infrastructural transformation, which often attract inward FDI, especially from the technologically advanced countries. We believe that the substantial inward FDI received by the GCC countries over the study period is attributed to their ongoing economic, urban periphery and sophisticated infrastructure developments in the last three decades.

## 6. Key recommendations

Based on the key findings presented and critically discussed above, some policy recommendations can be put forward to the relevant GCC authorities in their efforts to better understand crucial long-run determinants of FDI for their countries. These recommendations aim to enhance FDI inflows and stimulate economic growth. Depending on the similarity of the macroeconomic characteristics of GCC countries with those elsewhere in the world, these recommendations will likely have broader applicability.

### 6.1 Reducing constantly high unemployment

Not all, but most GCC countries face the challenge of constantly high unemployment rates, leading to a higher dependency on cheaper foreign workers for economic growth and prosperity. Therefore, it is essential for the GCC countries to explore ways to reduce their reliance on foreign workers and promote the employment of their domestic workforce. However, this task is not without its challenges, as employing a domestic workforce could potentially increase production costs, which might discourage some FDI. To address this issue, generating employment opportunities in economic sectors attractive to the domestic workforce and providing them with adequate training and financial incentives would be helpful in achieving this objective. By investing in the skills and capabilities of their own citizens, the GCC countries can build a more resilient and competitive labor force, making their economies more attractive to both domestic and foreign investors.

### 6.2 Maintaining population growth

No country can achieve sustainable economic growth without aligning it with its population growth. Population growth, especially workforce growth, plays a pivotal role in driving economic growth, which, in turn, is influenced by FDI. Population growth should never be perceived as a hindrance to economic growth, as higher population growth results in a larger workforce, leading to cost-effective production due to lower labor costs. This, in turn, attracts more FDI in the long run. The cause-and-effect relationship between higher population growth, an expanded workforce, lower labor costs, and increased FDI should be thoroughly understood by the GCC countries in their pursuit of attracting inward FDI. Embracing and efficiently utilizing their growing population can act as a catalyst for economic growth and further enhance the attractiveness of these countries as investment destinations.

### 6.3 Considering FDI as the driver of GDP growth

As mentioned earlier, the GCC countries are primarily growing by utilizing their vital non-renewable economic resources, making the case for attracting inward FDI crucial for economic resilience. Therefore, the GCC countries must explore ways to attract more inward FDI to enhance their economic resilience. Implementing favorable terms of trade, reducing taxes, removing existing and potential trade barriers, and establishing free trade agreements with major trading partners would all contribute to achieving this goal.

### 6.4 Continuing with infrastructure development and urbanization

Recent years have witnessed significant infrastructure development and rapid urbanization in most GCC countries, particularly in the UAE, Saudi Arabia, and Qatar. These infrastructure developments have attracted substantial inward FDI, prompting the GCC nations to improve their business infrastructures to make FDI even more attractive to the technologically advanced countries. The vast reserves of non-renewable resources in these countries have led to significant socioeconomic transformations, resulting in people migrating from remote regions to cities in search of better quality of life and improved public amenities. Consequently, rapid urbanization has become an inevitable outcome, and inward FDI has played a crucial role in facilitating such urbanization process. As a result, infrastructure development and urbanization should be viewed as conducive factors that highly attract inward FDI. The continuous growth and enhancement of infrastructure and urban areas in the GCC countries present attractive investment opportunities and contribute to their economic prosperity.

## 7. Conclusion

This study investigates the macroeconomic and environmental factors that could potentially attract inward FDI flows into the GCC countries, particularly in the long run. It also examines causality to determine how these factors affect FDI inflows into these countries. The factors that have been found to significantly attract inward FDI inflows into the GCC countries include economic growth, inflation, carbon dioxide emissions, and urbanization. Based on the findings, however, several policy recommendations have been provided. Aligning macroeconomic and environmental practices with these suggestions may effectively attract more inward FDI flows into the GCC countries.

Economic structural reform and free trade agreements beyond the GCC countries would facilitate the process of achieving the goal of attracting FDI into the GCC countries. Multinational corporations (MNCs), transnational corporations (TNCs), and small and medium-sized enterprises (SMEs) would consider investing in the GCC countries only when they find the socioeconomic, political, and legal environments favorable. Therefore, fostering both long-term economic incentives and creating conducive business infrastructures for investors are vital steps to attract inward FDI flows into any nation, including those in the GCC region.

Studies using time series data are always conducted with some limitations, which future studies may try to overcome. However, the perspectives of future studies lie in data availability to conduct similar research to see how these macroeconomic and environmental variables affect FDI inflows and outflows in other regions. Increase in sample size and conducting cross-regional studies are expected to provide wider picture of how FDI inflows and outflows are influenced by the macroeconomic and environmental variables in the GCC region and the beyond.
